# Pyrosequencing analysis of the protist communities in a High Arctic meromictic lake: DNA preservation and change

**DOI:** 10.3389/fmicb.2012.00422

**Published:** 2012-12-20

**Authors:** Sophie Charvet, Warwick F. Vincent, André Comeau, Connie Lovejoy

**Affiliations:** ^1^Département de Biologie, Université Laval, QuébecQC, Canada; ^2^Québec-Océan, QuébecQC, Canada; ^3^Centre d’études Nordiques, QuébecQC, Canada

**Keywords:** Arctic, anoxic, climate change, meromictic, phytoplankton, polar lakes, protists

## Abstract

High Arctic meromictic lakes are extreme environments characterized by cold temperatures, low nutrient inputs from their polar desert catchments and prolonged periods of low irradiance and darkness. These lakes are permanently stratified with an oxygenated freshwater layer (mixolimnion) overlying a saline, anoxic water column (monimolimnion). The physical and chemical properties of the deepest known lake of this type in the circumpolar Arctic, Lake A, on the far northern coast of Ellesmere Island, Canada, have been studied over the last 15 years, but little is known about the lake’s biological communities. We applied high-throughput sequencing of the V4 region of the 18S ribosomal RNA gene to investigate the protist communities down the water column at three sampling times: under the ice at the end of winter in 2008, during an unusual period of warming and ice-out the same year, and again under the ice in mid-summer 2009. Sequences of many protist taxa occurred throughout the water column at all sampling times, including in the deep anoxic layer where growth is highly unlikely. Furthermore, there were sequences for taxonomic groups including diatoms and marine taxa, which have never been observed in Lake A by microscopic analysis. However, the sequences of other taxa such as ciliates, chrysophytes, Cercozoa, and *Telonema *varied with depth, between years and during the transition to ice-free conditions. These seasonally active taxa in the surface waters of the lake are thus sensitive to depth and change with time. DNA from these taxa is superimposed upon background DNA from multiple internal and external sources that is preserved in the deep, cold, largely anoxic water column.

## INTRODUCTION

Meromictic lakes, with saline deep water overlain by fresh water, are known from both the north and south Polar Regions (Vincent et al., 2008b). Protists living in these perennially stratified environments encounter a range of extreme conditions. Those in the surface waters must tolerate cold temperatures, low nutrients, and reduced light due to the prolonged winter darkness and ice cover. The saline, usually anoxic, waters below the freshwater layer create another extreme environment. The pronounced vertical gradients in light, temperature, salinity, nutrients, oxygen, and other terminal electron acceptors provide a range of conditions for life within the same lake that could select for distinct communities down the water column.

Several meromictic lakes occur along the northern coast of Ellesmere Island, Canada, and were formed when seawater was trapped by isostatic uplift following the last de-glaciation and subsequent inflow of meltwater (Jeffries and Krouse, 1985). These lakes owe much of their continued water column stability to year-round ice cover and protection from wind-driven mixing (Vincent et al., 2008a). The deepest meromictic lake in the region, Lake A (83.00°N, 75.30°W), originated about 4000 years ago following the retreat of the Ellesmere Island glaciers (Jeffries and Krouse, 1985). The dense saline waters of the monimolimnion are separated from the surface fresh waters by a stable halocline. These deeper waters derived from the original seawater are mostly anoxic and would be predicted to harbor very different species compared to the freshwater surface layer (mixolimnion) originating from the surface runoff of catchment snowmelt that flows into the moat region and under the ice cover during summer (Veillette et al., 2012).

Previous studies of Lake A revealed that picocyanobacteria were abundant in the mixolimnion (Van Hove et al., 2008; Antoniades et al., 2009) with high concentrations of other bacteria in the deep monimolimnion, including green sulfur bacteria (Antoniades et al., 2009). Using 18S rRNA gene clone libraries and Sanger sequencing, Charvet et al. (2012) reported that the late summer protist communities in surface waters were dominated by chrysophytes and dinoflagellates, similar to Arctic non-meromictic lakes. Little is known, however, of the taxonomic makeup of protists down the water column (Veillette et al., 2011), with no previously published reports on the communities in the monimolimnion, and much of the protist diversity of this and similarly isolated far northern lakes remains unknown.

In the present study, we used high-throughput amplicon tag pyrosequencing of the V4 region of the 18S rRNA gene to examine protist communities down the water column. Specifically we compared communities from the surface mixed layer (the mixolimnion), the pycnocline and the saline deep layer (the monimolimnion). Cold, anoxic waters have been found to preserve extracellular DNA (e.g., Danovaro et al., 2005), and dead or dormant cells could accumulate in dense saline waters. These effects may create a background genetic signal that could mask the sequences from living organisms. Our aim was, therefore, to assess the extent of protist variations with depth and time relative to the background stocks of DNA that may be preserved in the deep waters of Lake A. Samples were collected from under the ice in spring (May 2008) and mid-summer (July 2009), and during a period of unusual warming and complete ice-out in late summer (August 2008). Since polar lakes are particularly vulnerable to ongoing climate change (Williamson et al., 2009), our results may indicate the type of community shifts that could follow the more regular loss of summer ice from these High Arctic ecosystems in the future.

## MATERIALS AND METHODS

### STUDY SITE, SAMPLING, NUTRIENTS, AND PHOTOSYNTHETICALLY ACTIVE RADIATION

Meromictic Lake A (83.00°N, 75.30°W) is located along the northern coast of Ellesmere Island, Nunavut, Canada. The surface area is 5 km^2^, with a drainage basin of 36 km^2^ and maximum depth of 128 m (Tomkins et al., 2009). Further details about this region are given in Vincent et al. (2011). Sampling was conducted on May 30, 2008; August 20, 2008; and July 20, 2009. In May and July, the lake was covered by 1.5–1.6 m of ice and 5–10 cm of snow; and in August 2008, it was exceptionally entirely free of ice. Physicochemical water column profiles were taken using a conductivity-temperature-depth (CTD) profiler (XR-420 CTD-RBR profiler; RBR Ltd, Ottawa, Canada). The potential density (sigma-theta) of the water was calculated using the *oce* package for the R program, based on the data for pressure, salinity, and temperature.

Water was collected at discrete depths with a Kemmerer bottle (Wildlife Supply Company, Yulee, FL, USA) and contents emptied directly into cleaned polypropylene containers after rinsing with sample water. Samples were collected from 2, 5, 10, 12, 20, 29, 32, and 60 m in May 2008; from 2, 10, 12, and 29 m in August 2008; and from 2, 5, 10, 12, and 29 m in July 2009. The ca. 12 L of collected water from each depth was kept cool, in the dark and transported back to a field laboratory within four hours. For DNA analysis, 3–4 L of water from the separate depths was sequentially filtered onto 47 mm diameter 3.0 μm pore size polycarbonate filters (Millipore) and 0.2 μm Sterivex units (Millipore). Lysis buffer (50 mM Tris, 40 mM EDTA, 0.75 M sucrose) was added to the cryovials containing the filters and to the Sterivex units, which were then stored at -80°C until DNA extraction.

Samples for nutrients were collected in 120 mL glass bottles with polypropylene caps and kept in the dark at ca. 4°C until analyses at the Canadian Center for Inland Waters (Burlington, Ontario. Concentrations of nitrate and nitrite (NOx) and soluble reactive phosphorus (SRP) were determined using standard colorimetric techniques (Gibson et al., 2002). The detection limit for NOx was 0.005 mg N L^–1^ and for SRP was 0.001 mg L^–1^.

Photosynthetically active radiation (PAR) values within the Lake A water column were derived from incident PAR data collected at Lake A in 2008 and 2009. Incident PAR was 53 and 33 mol photon m^–2^ day^–1^ in May and August 2008, respectively (Veillette et al., 2011), and 65 mol photon m^–2^ day^–1^ in July 2009. PAR immediately under the ice in May was estimated as described by Belzile et al. (2001). We estimated PAR levels at the sampled depths of our study in May and August 2008 and July 2009 (**Table 1**) based on the *albedo* and attenuation coefficient measurements from Belzile et al. (2001). The irradiance under the snow was calculated using the following equation:

**Table 1:** Estimates of irradiance within the water column, under the ice in May and directly under the surface in August 2008. *E*_z_ is in mol photons m^–2^ day^–1^.

	Depth (m)	Irradiance (*E*_z_					
		May 2008	Aug 2008	July 2009
0	Incident PAR	53	33	65
2	Under ice	0.38	–	1.03
	Under surface	–	14.97	–
5		0.132		0.359
10		0.022	0.896	0.062
12		0.011	0.443	0.031
20		0.0007	–	–
29		2.8 × 10^–5^	1.1 × 10^–3^	7.7 × 10^–3^
32		9.9 × 10^–6^	–	–
60		5.2 × 10^–10^	–	–

$$E_{\text{d(snow)}}=E_{\text{inc}}\times \left[1-\alpha_{\left(\text{snow}\right)}\right]\times {\text{e}}^{\left[-k_{\text{d}\left(\text{snow}\right)\times Z}\right]},$$

where *E*_inc_ is the incident irradiance at the surface of the snow, α is the *albedo* of the snow, *K*_d(snow)_ is the attenuation coefficient and *Z* the depth of the snow cover. The irradiance under the ice was obtained from the following equation:

$$E_{\text{d(ice) = }}\,E_{\text{d(snow)}}\times e^{\left[-k_{\text{d}\left(\text{ice}\right)\times Z}\right]},$$

where *K*_d(ice)_ is the attenuation coefficient of the ice and *Z* the depth of the ice cover. The irradiance at the different sampling depths of the water column was estimated from the following equation:

$$E_{z\text{ = }}\,E_{\text{d}(\text{surface})}\times e^{\left[-k_{\text{d}\left(\text{water}\right)\times Z}\right]},$$

where *E*_z_ is the irradiance at depth *Z*, *E*_d__(surface)_ is the irradiance at the surface, whether immediately under the ice or at the surface of the open water, and *K*_d(water)_ is the diffuse attenuation coefficient of the water column.

### CHLOROPHYLL *a* AND BIOMASS

Extracted chlorophyll *a* (Chl *a*) concentrations were derived from high performance liquid chromatography (HPLC) as detailed in Veillette et al. (2011) and Bonilla et al. (2005). Protist biomass was estimated from the light microscopy counts in Veillette et al. (2011). Taxon-specific biovolumes were calculated from the two dimensions noted either directly with an ocular micrometer or from images captured using a Qimaging Fast 2000R system (Qimaging, Surrey BC, Canada). Geometric differences between oblate spheres and ovoids, for example, were inferred from the literature. The biovolumes of more complex cell shapes were estimated following Hillebrand et al. (1999). Cell biovolumes were then transformed to carbon biomass (ng C L^–1^) based on the equations in Menden-Deuer and Lessard (2000).

### DNA EXTRACTIONS

Community DNA was extracted using a salt (NaCl) based method modified from Aljanabi and Martinez (1997) with lysozyme and proteinase K steps (Diez et al., 2001) as detailed in Charvet et al. (2012). The final ethanol-rinsed DNA pellets were dried and resuspended in 100 μL of 1× TE buffer (10 mM Tris–HCl, 1 mM EDTA) and stored at -80°C.

### PCR AMPLIFICATIONS AND SEQUENCING

Both large (3 μm) and small (0.2 μm) fractions from May and August 2008 were amplified separately then mixed in equal volumes for subsequent sequencing. Only the large fraction was amplified for July 2009 samples. The V4 region of the 18S rRNA gene was targeted with primers E572F and E1009R as described in Comeau et al. (2011). The V4 region is the longest variable region of the 18S rRNA gene and has relatively high taxonomic resolution (Dunthorn et al., 2012); even species can be distinguished within some groups, such as centric diatoms (Luddington et al., 2012). The forward primers included the Roche A adaptor and multiplex identifiers (MID-1 to -12) and the reverse primer included the Roche B adaptor. Amplicon DNA concentrations were measured using a Nanodrop ND-1000 spectrometer (Thermo Scientific, Wilmington, DE, USA) and equal quantities of the DNA from the individual samples were mixed and run on one eighth of a plate using the Roche 454 GS-FLX Titanium platform at the Plateforme d’Analyses Génomiques de l’Université Laval, at the Institut de Biologie Intégrative et des Systèmes, Québec, Canada. The raw reads were deposited in the NCBI Sequence Read Archive they are published under the accession number SRA057195.

### PRE-PROCESSING, QUALITY CONTROL, AND TAXONOMY ANALYSES

Raw sequence reads were initially filtered for unidentified nucleotides (Ns), bad primer and short reads (Comeau et al., 2011). Reads were randomly re-sampled to ensure the same number of reads for each MID tag, these were then pooled and aligned in Mothur against the SILVA reference alignment^1^ (Schloss et al., 2009) using the ksize = 9 parameter. Misaligned reads were removed at this point and aligned reads were clustered into operational taxonomic units (OTUs) at the ≥98% similarity level using furthest-neighbor clustering (Mothur). OTUs represented by only one sequence, singletons, may be part of the rare biosphere within a sample (Sogin et al., 2006), but may also arise from sequencing errors (Huse et al., 2010; Kunin et al., 2010). Our decision to discard these singleton-reads was therefore conservative, and the true diversity may be underestimated (Sogin et al., 2006). Read and OTU yields are presented in **Table 2**.

**Table 2:** Total sequence and OTU yields for each sample.

Sample	Depth (m)	Initial # reads	Clean # reads	Clean # OTUs
May	2	9498	4471	2073
	5	8381	4195	2431
	10	8646	4099	2366
	12	8201	4217	2275
	20	9158	4094	2294
	29	8285	3269	1990
	32	10609	3868	2446
	60	9946	3961	2433
August	2	8421	4014	1752
	10	9134	4208	2334
	12	8891	4067	2192
	29	9270	3612	2309
July	2	13368	4451	1062
	5	13716	4548	950
	10	14648	4813	861
	12	16438	4658	1041
	29	7960	4298	908

*The number of reads after equalization of samples was 5193; reads were binned into OTUs at 98% similarity. The “clean # reads” is the number reads left per sample after filtering badly aligned sequences and discarding singleton OTUs. “Clean # OTUs” is the final number of OTUs obtained from the clean number of reads. OTU, operational taxonomic unit.*

The taxonomic assignation was refined by assigning reads against our user-designed V4 reference sequence database using a 50% bootstrap cut-off. This reference database (available upon request) is based on the NCBI taxonomy database with added curated Arctic-specific sequences (Comeau et al., 2011) including those from Arctic lakes (Charvet et al., 2012). Common remaining “unclassified sequences” were further investigated using BLASTn (Altschul et al., 1990) against the GenBank nr database (NCBI).

### OTU-BASED ANALYSES

Communities from the different samples and depths were clustered using a Bray–Curtis analysis based on relative abundance of OTUs and using the Sorenson index based on presence-absence data (Mothur). Similarly, an un-weighted UniFrac analysis (Lozupone and Knight, 2005) was also carried out to take into account the fact that the samples from July only contained the 3 μm fractions of the communities, while the May and August samples had the 0.2 and 3 μm size fractions. An analysis of molecular variance (AMOVA) was also conducted (Mothur) to determine if there were significant differences among the communities of OTUs.

### STATISTICAL ANALYSES

The sequence abundance data were transformed to relative proportions before conducting multivariate analyses. A principal component analysis (PCA) was conducted on the physicochemical data (temperature, salinity, NOx, SRP, Chl *a*, and PAR). A canonical correspondence analysis (CCA) was performed to determine which environmental variables were correlated with changes among protist communities. We selected the most frequently occurring genera (representing ≥5% of the sequences belonging to a group) within the major protist groups representing ≥10% of the total sequences for at least one sample (ciliates, dinoflagellates, chrysophytes, diatoms, chlorophytes, Cercozoa, and *Telonemia*) to reduce the number of taxa used in the CCA. The PCA and CCA were performed using PAST software (Hammer et al., 2001). A correlation analysis was conducted in PAST on the environmental variables to avoid redundancy in the CCA, and none of the variables were significantly correlated, so all were kept for the ordination analysis. Evaluation of the significance of differences between the community structures, using the data at the genus level, was conducted with Metastats^2^ (White et al., 2009).

## RESULTS

### ENVIRONMENTAL PARAMETERS

**FIGURE 1:**
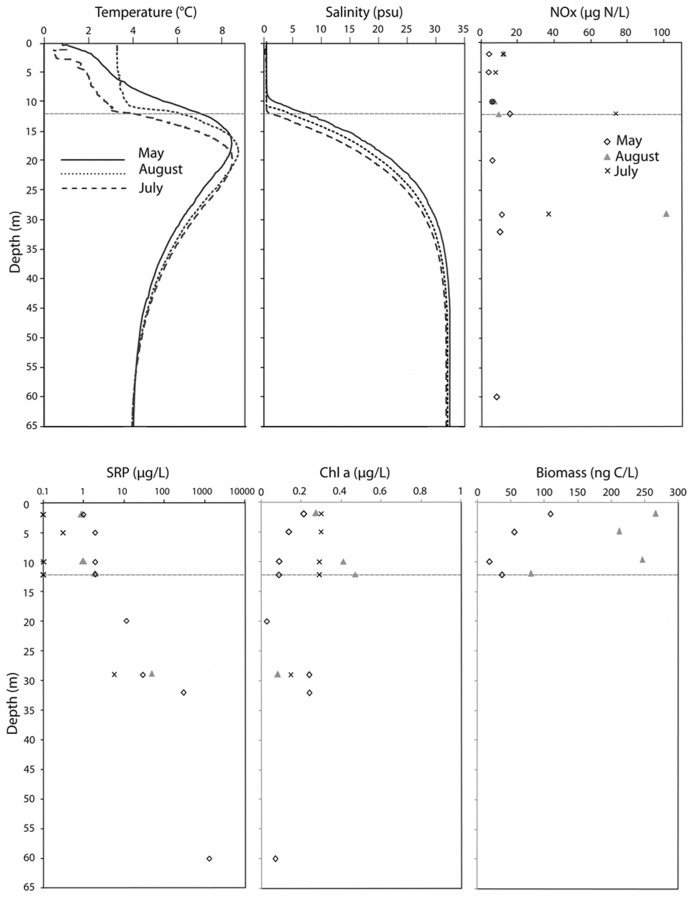
**Environmental variables of the Lake A water column in May 2008, August 2008 and July 2009**. NOx, nitrate and nitrite concentrations; SRP, soluble reactive phosphorus (note the logarithmic scale); Chl *a*, chlorophyll *a*. The depth axis is in meters.

The physicochemical profiles of Lake A, in May and August 2008, were previously reported by Veillette et al. (2011) and are summarized along with the July 2009 data (**Figure 1**). Salinity was lower at 12 m, in August 2008 and July 2009 compared to May 2008, indicating erosion of the halocline. The sigma-theta calculations reflected the strong stratification of the water column, with a two order of magnitude increase from 0.25 to 23 kg m^–3^, over the depth interval of 12 to 29 m. Nutrient concentrations (**Figure 1**) reflected the physical stratification, with much higher concentrations of SRP in the monimolimnion than in the freshwater mixolimnion. Chl *a* concentrations (**Figure 1**) were low, ranging from 0.03 to 0.48 μg L^–1^ and overall, greater in the mixolimnion in August and July compared to May (**Figure 1**). In July, the chl *a* concentrations were more homogenous throughout the mixolimnion with 0.29–0.30 μg L^–1^. Protist biomass increased in August compared to May by a factor of 2.5, and followed the same trends as Chl *a*, except at 12 m (**Figure 1**). At this depth, the biomass decreased while the Chl *a* concentration increased to reach its maximum concentration of 0.48 μg L^–1^.

### PROTIST COMMUNITIES

**FIGURE 2:**
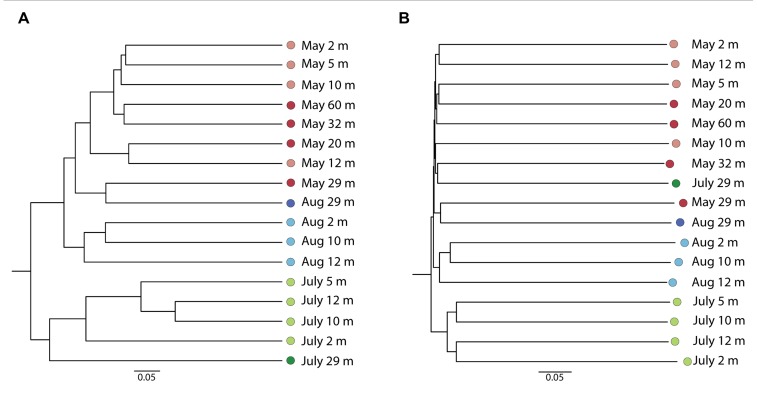
Bray–Curtis **(A)** and un-weighted Unifrac **(B)** dendrograms based on OTUs (98% similarity) from May 2008, August 2008, and July 2009.

**FIGURE 3:**
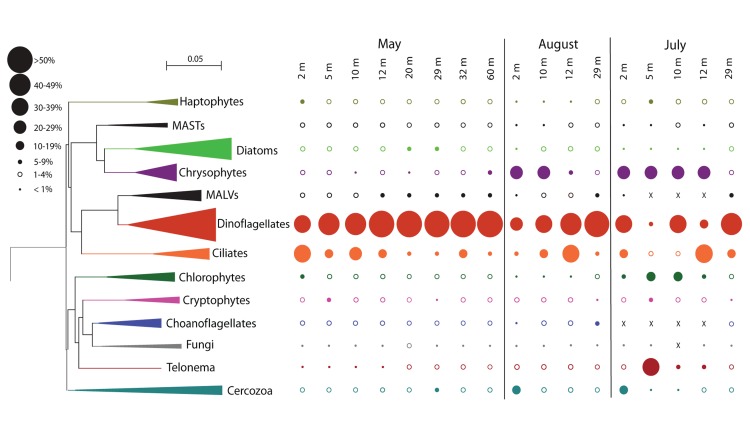
**Neighbor Joining eukaryote tree indicating the proportion of each phylum from the water column of Lake A at the three dates**. The size of the leaves is proportional to the number of genera within the groups from all samples. The sizes of the circles show proportions of sequence groups from each sample (scale in the upper left corner).

The Bray–Curtis clustering indicated a tendency of the communities to group by sampling date (**Figure 2A**) and the dendrogram obtained from the Sorenson index provided a similar clustering of samples (not shown). The May 2008 communities clustered together, except for May 29 m, which grouped with the August 29 m sample. The samples from July grouped apart from May and August 2008 samples. The un-weighted UniFrac dendrogram showed a similar separation of the communities by year (**Figure 2B**). However, the July 29 m sample grouped with the 32 m May 2008 sample. At the phylum level (**Figure 3**), no trends down the water column were evident, but given the Bray–Curtis and UniFrac clustering patterns, we investigated differences at finer taxonomic scales. Phyla were selected for detailed analysis on the basis of their particularly high sequence representation (dinoflagellates) and low variability (diatoms), or for their marked vertical and temporal changes (ciliates and chrysophytes).

**FIGURE 4:**
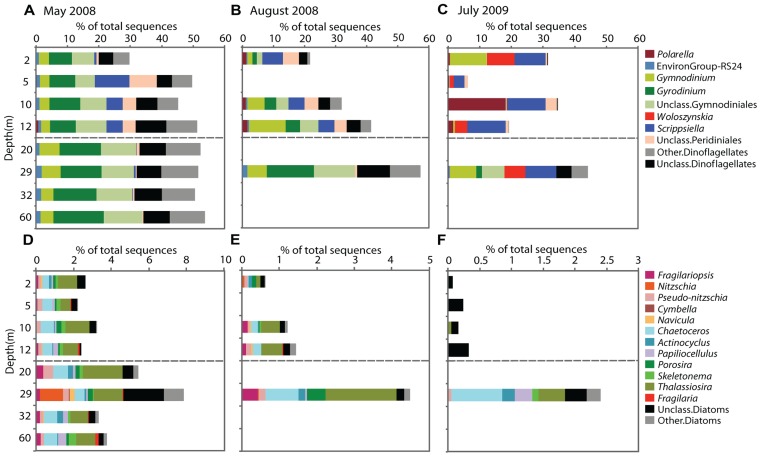
**Sequences of genera showing seasonal stability within Lake A in May 2008, August 2008, and July 2009**. The bar graphs represent the dinoflagellate **(A–C)** and diatom genera **(D–F)**. Mixolimnion depths are above the dashed line and monimolimnion depths below. The proportions are based on total number of sequences (note differences in the *x*-axis scale for the different sampling times and groups).

The Dinophyceae accounted for the greatest proportion of sequences throughout the water column in May, contributing 30–50% of sequences in the mixolimnion and > 50% in the monimolimnion (**Figure 3**). At the genus level, *Scrippsiella* and unclassified Peridiniales sequences were recovered from the mixolimnion but not the monimolimnion (**Figure 4A**). In August, the overall proportion of dinoflagellates was less but the relative proportion of *Scrippsiella* sequences was greater compared to May. Sequences with best matches to *Polarella* were also recovered in the August mixolimnion (**Figure 4B**). In July 2009, the relative dinoflagellate abundance varied, representing > 30% at 2, 10, and 29 m, and 7 and 19% at 5 and 12 m, respectively. Genera also varied with depth, with increased proportions of *Scrippsiella*, the appearance of the freshwater genus *Woloszynskia* and the dominance of *Polarella* sequences at 10 m (**Figure 4C**).

Diatoms dominated the stramenopile sequences (38–80%) in May, representing 4% of total protist sequences in the mixolimnion and up to 9% in the monimolimnion (**Figure 3**). There was little taxonomic change among depths (**Figure 4D**). Diatom proportions were lower in August, with <2% in the mixolimnion (**Figure 4E**). However, at 29 m diatoms represented 4% of the total sequences and accounted for 50% of the stramenopile sequences. In July, the diatoms represented <0.5% of the total sequences from the mixolimnion and a BLAST analysis against the GenBank nr database showed that most of those sequences were closest to (95–97% similarity) to uncultured freshwater environmental sequences (**Table 3**). At 29 m, the diatom sequences represented close to 2.5% of total sequences (**Figure 4F**).

**Table 3:** BLAST search results for unclassified diatom sequences in July 2009 samples.

Depth	Seq	Closest match	%	Acc. #	Origin	Reference
2	3	Unc. freshwater clone LG22-09	96	AY919761	Adirondack Park, USA. Lake George	Richards et al. (2005)
		*Bolidomonas mediterranea* CCMP:1867	89	HQ710555	Culture	Yang et al. (2012)
5	3	Unc. freshwater clone LG22-09	97	AY919761	Adirondack Park, USA. Lake George	Richards et al. (2005)
		*Bolidomonas mediterranea* CCMP:1867	89	HQ710555	Culture	Yang et al. (2012)
10		Unc. freshwater clone LG22-09	96	AY919761	Adirondack Park, USA. Lake George	Richards et al. (2005)
		*Biddulphia alternans* ECT3856	91	HQ912677	Culture	Theriot et al. (2010)
12	1	Unc. freshwater clone LG22-09	95	AY919761	Adirondack Park, USA. Lake George	Richards et al. (2005)
		*Biddulphia alternans* ECT3856	90	HQ912677	Culture	Theriot et al. (2010)
	7	Unc. freshwater clone LG22-09	96	AY919761	Adirondack Park, USA. Lake George	Richards et al. (2005)
		*Bolidomonas mediterranea* CCMP:1867	89	HQ710555	Culture	Yang et al. (2012)
	2	Unc. freshwater clone LG22-09	97	AY919761	Adirondack Park, USA. Lake George	Richards et al. (2005)
		*Bolidomonas pacifica*	90	AB430618	Culture	Sato et al., Unpublished
29	1	Unc. *Chaetoceros* clone MALINA_St390_3m_Pico_ES020_P1H10	99	JF698751	Beaufort Sea, Canada. 3 m depth	Balzano et al. (2012)
		*Chaetoceros decipiens* strain RCC1997	93	JF794044	Culture	Balzano et al. (2012)
	9	Unc. marine picoeukaryote ws_101, clone 1807E08	97	FR874617	Norwegian fjord. Marine coastal water	Newbold et al. (2012)
		*Chaetoceros decipiens* strain RCC1997	91	JF794044	Culture	Balzano et al. (2012)

*The first name listed was the closest BLAST match, the second name listed was the closest cultured match. Depth in meters; Seq, number of sequences; %, percent similarity; Acc.#, NCBI accession number; Origin, location from which the sequence was obtained; Unc., uncultured.*

**FIGURE 5:**
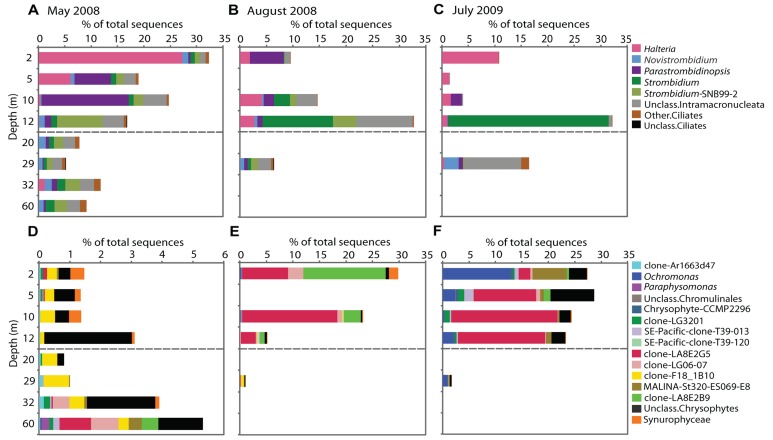
**Sequences of genera showing seasonal changes within Lake A in May 2008, August 2008 and July 2009**. The bar graphs represent the ciliates **(A–C)** and chrysophyte genera **(D–F)**. Mixolimnion depths are above the dashed line and monimolimnion depths below. The proportions (note differences in the *x*-axis scale for the different sampling times and groups) are based on total number of sequences.

In May 2008, the ciliate sequences were proportionally more abundant in the mixolimnion, especially at 2 m, and fewer deeper down the water column (**Figure 3**). The most commonly represented genera were *Halteria*, *Parastrombidinopsis*, and *Strombidium* and the proportional representation of these taxa varied with depth (**Figure 5A**). In May, the ciliate community at 12 m resembled the underlying monimolimnion rather than that in the mixolimnion. In August, a change in relative representation of ciliates was observed with ciliates accounting for a greater proportion of sequences at the bottom of mixolimnion (12 m; **Figure 3**) with a relative increase of *Strombidium* sequences (**Figure 5B**). In July 2009 *Halteria* was again common at 2 and 5 m, while sequences related to *Strombidium *were mostly at 12 m (**Figure 5C**). At 29 m, in May 2008 ciliates were diverse, whereas in July 2009 novel currently unclassified ciliate sequences had highest representation (**Figure 5C**).

Within the overall May 2008 protist community, stramenopile sequences represented 5–12%, of which 7–40% were assigned to chrysophytes (**Figure 3**). These chrysophyte sequences (**Figure 5D**) were mostly either novel or related to uncultured environmental 18S rRNA clones (Richards et al., 2005; Behnke et al., 2006; Scarcella, 2009; Charvet et al., 2012). In May, the majority of chrysophyte taxa were restricted to either the mixolimnion or monimolimnion (**Figure 5D**), with a few exceptions, such as those classified with clones FV18_1B10 (Behnke et al., 2006) and Ar1663d47 (Scarcella, 2009). From May to August, the proportion of chrysophyte sequences in the mixolimnion increased from 1% to 24–29% of the total (**Figure 3**), with a taxonomic change, including the appearance of sequences that matched several clones previously retrieved from Lake A, such as LA8E2G5 (**Figure 5E**). The July 2009 relative proportions of chrysophytes were comparable to those of August 2008 (**Figure 3**), but with some differences in the community, as other chrysophyte taxa were recovered in addition to LA8E2G5, such as *Ochromonas*-related sequences (**Figure 5F**).

From May to August 2008, and to July 2009, as the proportion of dinoflagellates and ciliates decreased, the protist community was also marked by increases in the proportion of sequences associated with other strict heterotrophic groups (**Figure 3**). The Cercozoa represented <2% of total protist sequences in the mixolimnion in May 2008, but in August 2008 and July 2009 this group represented 13 and 17%, respectively at 2 m. The proportions of *Telonema* sequences also increased, rising from <1% in May to 1–4% in August, and reaching ~40% at 5 m in July 2009. At the later date, *Telonema* actually dominated the heterotroph community at 5 m, as Cercozoa, ciliates and dinoflagellate sequences were reduced to 0.7, 1.5, and 6%, respectively.

### STATISTICAL AND ORDINATION ANALYSES

At the OTU level, the May mixolimnion (2, 5, 10, and 12 m) and monimolimnion (20, 29, 32, and 60 m) were not significantly different (AMOVA, *F*s = 1.12, *p* = 0.285). In contrast, the August and July communities in the mixolimnion and those at 29 m were highly significantly different from each other (*F*s = 2.56, *p* < 0.001 and *F*s = 1.52, *p* < 0.001, respectively) and the mixolimnion communities of May were also significantly different from those at 29 m at the same date (AMOVA, *F*s = 1.92, p = 0.048). The mixolimnion communities from each date had significantly distinct OTU compositions (AMOVA, *F*s_(May/Aug)_ = 2.12, *F*s_(May/July)_ = 4.7, *F*s_(Aug/July)_ = 3.7, *p* < 0.001) which was also reflected in the community structure at the genus level. Compared with the mixolimnion in May 2008 the communities in August 2008 and July 2009 were significantly (Metastats, *p* < 0.05) enriched in some genera.

**FIGURE 6:**
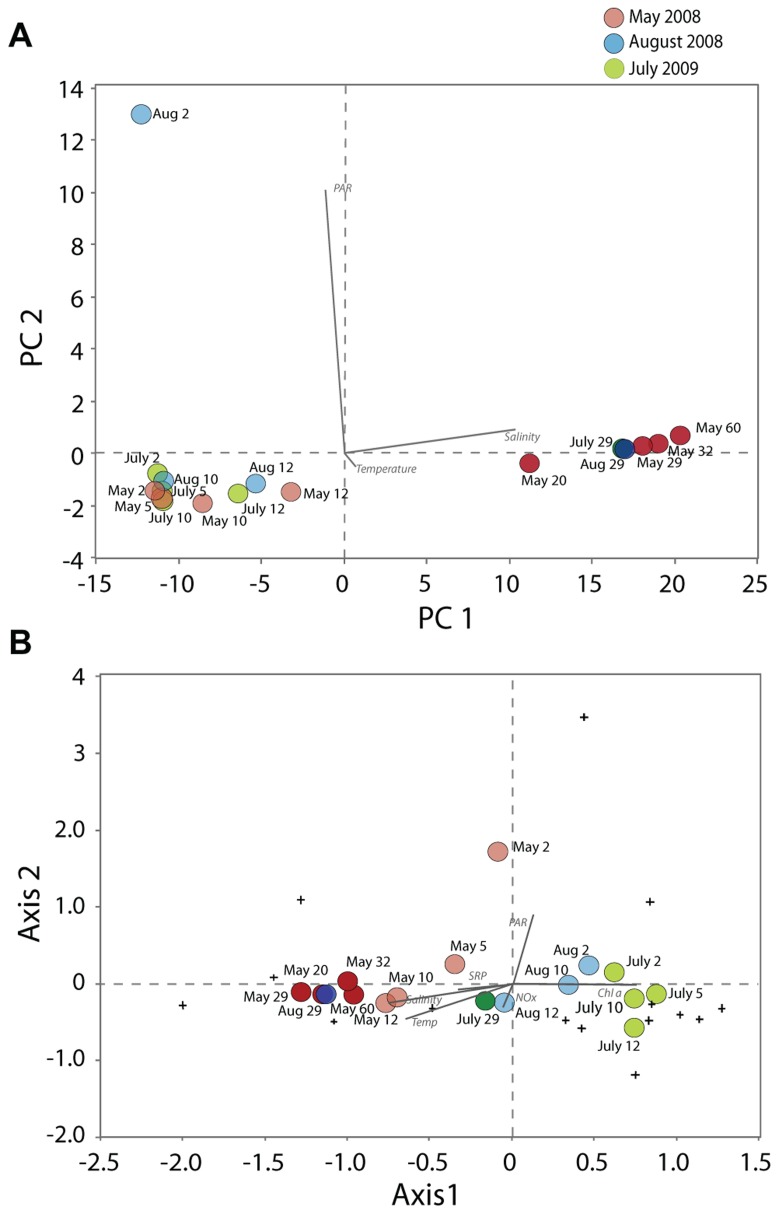
Principal component analysis **(A)** based on non-transformed environmental variables (temperature, salinity, PAR, NOx, SRP, chlorophyll *a*), and canonical correspondence analysis **(B)** using sequence proportions of all genera and the same environmental variable. Samples from the mixolimnion are represented in light hues and from the monimolimnion in dark hues. Samples of May 2008 are in red, August 2008 in blue and July 2009 in green. Note that the loadings for the nutrient and chlorophyll *a* variables are not indicated in the tri-plot **(A)** because they were not significantly different from zero.

A PCA with the environmental variables including temperature, salinity, NOx, SRP, Chl *a*, and PAR (eigenvalues 73.9% for PC1 and 25.5% for PC2) showed the abiotic segregation of samples (**Figure 6A**). This PCA showed that samples mostly grouped according to depths and water column strata, along the gradients of salinity (loading of 0.99 along Axis 1) and PAR (loading of 0.99 along Axis 2). A CCA with the abundance data of the dominant genera of the most variable groups (dinoflagellates, ciliates, chrysophytes, diatoms, Cercozoa, *Telonema*, chlorophytes) of May, August and July, using the same environmental variables, revealed that adding biological parameters caused a different pattern of segregation in ordination space (**Figure 6B**; eigenvalues of Axis 1 and 2 were 49.48 and 28.8%, respectively). The communities were distributed according to date and salinity, Chl *a*, temperature, and PAR seemed to be the most influential factors in structuring the DNA-inferred protist composition in Lake A. A CCA was also conducted based on a presence-absence matrix (not shown), and provided similar results. The separation of samples according to date was even more accentuated, with stronger similarity between May samples from the mixolimnion and the monimolimnion.

## DISCUSSION

### DNA PRESERVATION AND CONSTANCY

Lake A is strongly meromictic with anoxic bottom waters likely persisting since it was formed several thousand years ago. The presence of banded iron deposits in the sediments of the lake indicates that only brief intervals of oxycline erosion have occurred in the past (Tomkins et al., 2009). The large differences between the mixolimnion and monimolimnion nutrient, oxygen and salinity conditions in Lake A are typical of meromictic lakes (Lauro et al., 2011). The major ion content of the water column (Na^+^, K^+^, Mg^2+^, Ca^2+^, Cl^–^, ${\text{SO}}_{4}^{2-}$, ${\text{CO}}_{3}^{2-}$, ${\text{HCO}}_{3}^{-}$) was analyzed in Gibson et al. (2002). The authors found that the surface waters (mixolimnion) contained a higher proportion of Ca^2+^ and Mg^2+^ in the cations compared to the deeper waters (monimolimnion), which were enriched in Na^+^, indicative of their marine origins. In May 2008, Lake A temperature and salinity profiles were very similar to those previously published (Hattersley-Smith et al., 1970; Belzile et al., 2001; Van Hove et al., 2001, 2006) reflecting the physical stability of the system and lack of wind mixing (Vincent et al., 2008a). In August 2008, the surface of the lake was exposed to wind driven mixing and there was evidence that the halocline had been slightly eroded (Veillette et al., 2011). Despite these persistent vertical gradients in habitat properties, the proportional abundance of protist DNA sequences showed only muted shifts down the water column, particularly at the phylum level.

The relative constancy with depth of most protists identified from the DNA contrasts markedly with a parallel study of the prokaryotes in Lake A, also using high-throughput sequencing. This latter analysis of 16S rRNA genes indicated that both archaeal and bacterial communities in Lake A are very different even at the level of phylum in the mixolimnion and monimolimnion, with typical anaerobic groups in the deeper waters (Comeau et al., 2012). At a finer taxonomic level based on OTUs defined at a level of 97%, seasonal changes in the bacterial community were also evident in the mixolimnion between May and August. The changes in the bacterial communities suggest strong environmental selection and community turnover at least in the surface waters.

The lack of marked variation of the eukaryotic community sequences implies a high background of either inactive encysted cells or recalcitrant DNA. The combination of cold, saline, and anoxic conditions in the water column likely ensure a certain level of preservation of extracellular DNA, both autochthonous and allochthonous. Of particular note, diatom communities even at the level of genera were similar down the Lake A water column, and they represented a larger proportion of the total protist sequences in the monimolimnion. It is doubtful that the diatoms were active in the suboxic and sulfidic zones, but rather that sequences detected with DNA were from sedimented dead or dormant cells, accumulated and preserved in the lake’s cold salty waters, as has been found in analogous cold, saline habitats elsewhere (Danovaro et al., 2005; Nielsen et al., 2007; Borin et al., 2008; Terrado et al., 2012). Similarly, the dinoflagellate genera found in the May and August 2008 profiles changed little with depth, with the exception of *Scrippsiella *and unclassified Peridiniales sequences that were only present in the mixolimnion, where they would be expected to be active. The remaining dinoflagellate genera were recovered irrespective of depth, suggesting that most sequences detected were not from an active community since genera composition would be expected to change with vertical and temporal shifts in light, nutrients, and oxygen levels. Dinoflagellates have high copy-numbers of ribosomal genes (Zhu et al., 2005) and were likely over-represented. Therefore, the relative proportion of dinoflagellate sequences does not directly reflect the proportion of cells or their level of activity. In addition, there was no report of diatoms and few dinoflagellate counts (≤2 × 10^3^ cells L^–1^) in a microscopy study carried out in parallel with our study (Veillette et al., 2011). The following year, there were marked differences in the diatom and dinoflagellate genera detected in all samples, relative to 2008. Interestingly, many of the diatom genera are considered polar marine species (Lovejoy et al., 2002) suggesting, firstly, that marine particles reach the lake either by way of transport in snow or directly by air (Harding et al., 2011) and, second, that there are interannual differences in the input of material into the lake. To correct for the detection of non-active or passively transported phylotypes that are inherent to DNA-based sequencing (Pawlowski et al., 2011) and as discussed above, we have limited the remaining discussion to genera or groups that varied over depths and seasons. Among these were the ciliates, chrysophytes, Cercozoa, and *Telonemia*. Another caveat is that the number of copies of the 18S ribosomal RNA gene per cell varies greatly among taxa, and we can therefore only compare the relative contributions of each group to the total number of sequences among depths and dates.

### WATER COLUMN DISTRIBUTIONS IN MAY 2008

Ciliates in May 2008 surface waters were related to the freshwater genus *Halteria* and in the lower mixolimnion (10 m) were related to *Parastrombidinopsis*. The small ciliate *Halteria grandinella* is an efficient grazer on picoplankton (Peŝtová et al., 2008).). *Halteria* was detected in the top 5 m of the mixolimnion in the fresher and colder waters directly under the ice (Veillette et al., 2011), where photosynthetic picocyanobacteria are most abundant (Van Hove et al., 2008). Chrysophytes made up a small proportion of the May sequences and were characterized by the presence of Synurophyceae especially *Mallomonas.* This silica-scaled chrysophyte is reported to have low potential for dispersal (Kristiansen, 2007) and was mostly in the freshwater surface from 2 to 10 m. In May 2008, the mixolimnion was limited to the upper 10 m and the 12 m halocline communities were physically isolated from those above. The chrysophyte communities at 12 m contained significant proportions of unclassified sequences. A BLASTn search of the GenBank database (**Table 4**) indicated that the majority of these sequences grouped with environmental clone 1815H10 from a coastal Norwegian fjord (FR874767; Newbold et al., 2012), suggesting a halotolerant species consistent with the brackish conditions at 12 m in May. Among ciliates, the genus *Strombidium* is a diverse genus with over 100 described species and is found in a variety of habitats (Wylezich and Jürgens, 2011). An uncultivated species with broad ecological tolerances could account for its presence at 12 m as well as in the deeper monimolimnion waters.

**Table 4:** BLAST search results for unclassified chrysophyte sequences in the 12 m sample from May 2008.

Seq	Closest match	%	Acc. #	Origin	Reference
14	Unc. freshwater eukaryote K5MAR2010	97	AB622322	Gunma, Japan. Freshwater lake Kusaki	Fujimoto, unpublished data
	*Paraphysomonas foraminifera*		Z38025		Rice et al. (1997)
17	Marine picoeukaryote ws_159, clone 1815H10	98	FR874767	Marine biome, fjord, coastal water	Newbold et al. (2012)
7	Unc. Eukaryote clone CYSGM-8	97	AB275091	Sagami Bay, Japan. Methane cold seep sediment	Takishita et al. (2007)
	*Oikomonas* sp. SA-2.1		AY520450	South Africa	Cavalier-Smith and Chao (2006)
9	Unc. stramenopile clone 5c-F12	97	FN690679	Bothnian Bay, Sweden. Sea ice	Majaneva et al. (2011)
	*Oikomonas* sp. SA-2.1		AY520450	South Africa	Cavalier-Smith and Chao (2006)
5	Unc. freshwater eukaryote K7MAY2010	96	AB622338	Gunma, Japan. Freshwater Lake Kusaki	Fujimoto, unpublished data
	*Spumella*-like flagellate JBM08		AY651098	Austria. Lake Mondsee	Boenigk et al. (2005)
5	Unc. freshwater eukaryote K7MAY2010	97	AB622338	Gunma, Japan. Freshwater Lake Kusaki	Fujimoto, unpublished data
	*Paraphysomonas foraminifera*		Z38025		Rice et al. (1997)
1	Unc. eukaryote clone CYSGM-8	97	AB275091	Sagami Bay, Japan. Methane cold seep sediment	Takishita et al. (2007)
	*Oikomonas* sp. SA-2.1		AY520450	South Africa	Cavalier-Smith and Chao (2006)
1	Unc. freshwater eukaryote clone LG26-08	97	AY919776	Adirondack Park, USA. Lake George	Richards et al. (2005)
	*Paraphysomonas foraminifera*		Z38025		Rice et al. (1997)
1	Unc. marine eukaryote clone MIF_CilE6	96	EF526986	Framvaren Fjord, Norway	Behnke et al. (2010)
	*Spumella* sp. 9-12-B3		EU787418		Chatzinota et al., unpublished
1	Unc. freshwater eukaryote clone LG26-08	95	AY919776	Adirondack Park, USA. Lake George	Richards et al. (2005)
	*Paraphysomonas foraminifera*	**	Z38025		Rice et al. (1997)

*The first name listed is the closest BLAST match, the second name listed is the closest cultured match. Depth, in meters; Seq, number of sequences; %, percent similarity; Acc.#, GenBank accession number; Origin, location from which the sequence was obtained; Unc., Uncultured.*

The chemocline of Lake A is suboxic and extends from approximately 13 to 20 m where the water becomes anaerobic, and the sulfidic zone starts at 32 m (Gibson et al., 2002). Protist DNA is often recovered from such extreme habitats (Edgcomb et al., 2009), including chrysophytes and ciliates (Wylezich and Jürgens, 2011; Orsi et al., 2012; Stock et al., 2012). In the present study, there were relatively fewer ciliate sequences in the chemocline and monimolimnion, compared to upper waters. Chrysophyte sequences were found throughout the chemocline, including sequences related to the clone FV18_1B10, from the super-sulfidic anoxic Framvaren Fjord (Behnke et al., 2010).

Ciliates prey on both phytoplankton (Pedrós-Alió et al., 1995) and bacteria (Saccà et al., 2009) in anoxic monimolimnia. Maximum bacterial pigment concentrations in Lake A are between 25 and 30 m, with the highest bacterial densities between 27.5 and 29 m (Antoniades et al., 2009). Photosynthetic sulfur bacteria live on the sulfides diffusing from the sulfidic zone under 32 m (Sakurai et al., 2010). The purple-sulfur bacteria are found in deeper layers than the green-sulfur bacteria (Comeau et al., 2012) and both could be grazed by ciliates (Wylezich and Jürgens, 2011) able to live under anaerobic conditions (Muller, 1993; Edgcomb et al., 2011). The significant unclassified Intramacronucleata ciliates from 29 m in May had a best BLAST match (97% similarity) to an Alveolate clone 5b-D8 from Baltic Sea ice in Bothnian Bay, Sweden (Majaneva et al., 2011); the closest match to an anoxic sourced clone (95% similarity) was to the eukaryote clone cLA12B10 (EU446380) from the halocline of the anoxic hypersaline l’Atalante basin (Alexander et al., 2009), suggesting that it belongs to either a cold adapted or anoxic species. Additional sequences from more environments and cultivated anoxic strains are required to clarify these affinities.

### TEMPORAL VARIATION

The ciliate and chrysophyte communities showed clear changes among the three dates. There was a remarkable shift from *Halteria* to *Parastrombidinopsis* in the mixolimnion between May and August, and a reoccurrence of *Halteria* in July 2009, especially at 2 m.* Halteria *is a small, fast-swimming (Ueyama et al., 2005) bacterivorous ciliate (Simek et al., 2000), while *Parastrombidinopsis *is a large marine brackish choreotrich (Agatha, 2011) that feeds on large protists such as dinoflagellates and diatoms (Kim et al., 2005; Tsai et al., 2008). These results suggest the availability of larger prey under the August conditions compared to May or July when the lake was ice-covered. Interestingly, the maximum proportion of ciliate sequences was found immediately under the ice at 2 m in May 2008, while in August 2008 and July 2009 the peaks were at 12 m. Consistent with the ciliate co-occurring with their favored food sources, Chl *a* concentrations followed the same pattern with maxima at 2 m in May 2008 and at 12 m in August 2008 (Veillette et al., 2011) although they were uniform from 2 to 12 m in July (this study).

The chrysophyte community changed between May, August 2008 and July 2009, with greater vertical differences in August and July compared to May. Although chrysophytes accounted for a low proportion of total sequences in May, they were sensitive to environmental changes and accounted for higher proportions of the total community in August 2008 and July 2009. The August surface water community was previously investigated with microscopy, pigment analysis, and clone libraries (Veillette et al., 2011; Charvet et al., 2012). The pronounced increase in chrysophytes seemed to be most closely related to the increased PAR availability, as was shown in the CCA. The sequences that classified with Clone LA8E2G5 (Charvet et al., 2012) represented the dominant chrysophyte group of the mixolimnion of August and July in the present study. Phylogenetic analysis indicated that this clone grouped within a clade represented by *Kephyrion *(Charvet et al., 2012), which was consistent with the microscopy (Veillette et al., 2011). The other dominant chrysophyte sequence in August 2008 was matched to clone LA8E2B9, which grouped at the base of the chrysophyte phylogenetic tree (Cluster I in Charvet et al., 2012). In July 2009, the chrysophytes were dominated by *Ochromonas *sequences, at 2 m, while the rest of the mixolimnion still contained sequences of the putative *Kephyrion *sp. clone LA8E2G5. Chrysophytes are nanoflagellates with the capacity for motility and can therefore maintain their position in the water column (Pick and Lean, 1984), such as in the surface waters where PAR would be most available.

The comparison of community composition over the three dates revealed differences in the presence of other bacterivores. Many of these heterotrophs were found throughout the water column in May and assumed to be mostly non-active or background DNA. However, they represented higher proportions of the sequence totals in August and July suggesting active growth. They occurred mostly in surface waters but not in deeper waters on those dates. In particular, Cercozoa were well represented at 2 m in August and July, but not in May, while *Telonema *dominated the protist sequences in July at 5 m, mostly replacing the dinoflagellate and ciliate sequences. Nonetheless, the dinoflagellate communities of July 2009 were less homogenous throughout the water column than in 2008, indicating that the sequences were more likely from active cells reacting to the environmental conditions at each depth. For example, *Polarella* was found at 10 m in July, and this genus was originally isolated from sea ice (Montresor et al., 1999), but has been reported from marine influenced, meromictic lakes in Antarctica (Rengefors et al., 2008). The salinity within sea ice can vary and sea ice associated species are often euryhaline, and it is possible that *Polarella* may be viable in the mixolimnion. Interestingly, we have been able to maintain a culture of Arctic *Polarella* (strain CCMP2088) in low salinity water over several weeks (Charvet and Lovejoy, unpublished data).

The changes in relative sequence abundance of protist taxa in the Lake A mixolimnion between May and August contrast with reports from Lake Fryxell. This perennial ice covered lake is located at a similar latitude in Antarctica (77.37°S for Lake Fryxell; 83.00°N for Lake A), yet the authors report no pattern of seasonal succession, but rather an increase in population densities of all species that were present at the beginning of the growing season (Spaulding et al., 1994). This contrasting response would be consistent with the large scale shifts in the mixolimnion environment of Lake A associated with ice-out between May and August 2008, for example the 40-fold increase in light availability and direct wind-induced mixing (Veillette et al., 2011). Furthermore, the PCA and CCA indicated that PAR was a determining factor for the biological changes between May, August, and July. The 29 m community showed little change during this period, consistent with this being a zone of dead protist accumulation rather than growth. Even after ice-out there would be little light, much less than 1% surface irradiance, for phototrophic protists at this depth (Belzile et al., 2001).

The two ice-covered sampling dates showed evidence of interannual variability in overall protist composition, although this may also reflect seasonal changes between May and July. In a previous study based on pigment data (Antoniades et al., 2009), the monimolimnion phototrophic communities (dominated by green sulfur bacteria) were similar from year to year, whereas the mixolimnion communities were more variable. Our results showed that the 29 m protist communities separated according to year, implying greater interannual variability in the anoxic zone for eukaryotes than for photosynthetic bacteria. Extreme variability between years has been reported for Lake Fryxell, where each year over 5 years a different phototroph dominated the phytoplankton (Spaulding et al., 1994). This variability has been attributed in part to differences in overwintering populations and to differences in stream flow inputs of nutrients, DOC and algal inocula between years (McKnight et al., 2000). Similar factors may influence the year-to-year differences in protist community structure in Lake A.

## CONCLUSION

Our high-throughput analysis of the Lake A protist community indicated the preservation of DNA throughout the water column. This relatively constant background included taxa derived from external sources such as diatoms and marine dinoflagellates that are unlikely to be active in the lake, particularly in the anoxic monimolimnion. There were pronounced changes in the upper water column that were superimposed upon this background, particularly in the mixolimnion between the late winter ice-covered period in May 2008 and the unusual open water conditions in late summer, August 2008. These results underscore the need for discrimination between active and inactive components of protist communities, for example direct sequencing of ribosomal RNA as cDNA or targeted mRNA sequencing to detect gene expression. Nevertheless, our approach was sufficiently sensitive to detect change and provides a baseline to gage the potentially larger changes in protist community structure that may occur with accelerated warming and ice loss in the High Arctic.

## Conflict of Interest Statement

The authors declare that the research was conducted in the absence of any commercial or financial relationships that could be construed as a potential conflict of interest.

## Footnotes

1 http://www.mothur.org/wiki/Silva_reference_files

2 http://metastats.cbcb.umd.edu
